# Impacts of invasive fish removal through angling on population characteristics and juvenile growth rate

**DOI:** 10.1002/ece3.1471

**Published:** 2015-05-09

**Authors:** Charlotte Evangelista, Robert J Britton, Julien Cucherousset

**Affiliations:** 1 CNRS, Université Toulouse III Paul Sabatier, ENFA, UMR5174 EDB (Laboratoire Évolution & Diversité Biologique)118 route de Narbonne, Toulouse, F-31062, France; 2 Departement of Life and Environmental Sciences, Faculty of Science and Technology, Bournemouth UniversityPoole, Dorset, BH12 5BB, UK

**Keywords:** Biological invasions, invasive species management, *Lepomis gibbosus*, nonrandom selection, recreational angling

## Abstract

Exploitation can modify the characteristics of fish populations through the selective harvesting of individuals, with this potentially leading to rapid ecological and evolutionary changes. Despite the well-known effects of invasive fishes on aquatic ecosystems generally, the potential effects of their selective removal through angling, a strategy commonly used to manage invasive fish, are poorly understood. The aim of this field-based study was to use the North American pumpkinseed *Lepomis gibbosus* as the model species to investigate the consequences of selective removal on their population characteristics and juvenile growth rates across 10 populations in artificial lakes in southern France. We found that the maximal individual mass in populations decreased as removal pressure through angling increased, whereas we did not observed any changes in the maximal individual length in populations as removal pressure increased. Total population abundance did not decrease as removal pressure increased; instead, here was a U-shaped relationship between removal pressure and the abundance of medium-bodied individuals. In addition, population biomass had a U-shaped curve response to removal pressure, implying that invasive fish populations can modulate their characteristics to compensate for the negative effects of selective removals. In addition, individual lengths at age 2 and juvenile growth rates decreased as removal pressure through angling increased, suggesting a shift toward an earlier size at maturity and an overall slower growing phenotype. Therefore, these outputs challenge the efficiency of selective management methods, suggesting the use of more proactive strategies to control invasive populations, and the need to investigate the potential ecological and evolutionary repercussions of nonrandom removal.

## Introduction

Invasive species are recognized as a major driver of global change that can invoke major ecological, evolutionary, and economic consequences (Pimentel et al. 2005). Freshwater ecosystems are particularly vulnerable to biological invasions, with non-native fish being introduced through a variety of pathways (e.g., aquaculture, fisheries, aquarium trade) and incurring ecological impacts across different levels of biological organization (Gozlan et al. 2010; Cucherousset and Olden 2011). Where introductions of non-native fish result in invasions, their management is inherently difficult and often limited to removals via targeted captures that are commonly performed through fishing, including angling (Britton et al. 2011). While the potential efficiency of methods used to decrease the stock of invasive populations has been reported (Cucherousset et al. 2006a; Britton et al. 2011), the removal of individuals is often selective (Coltman et al. 2003; Wilson et al. 2011) and might lead to counterproductive results, including an increased abundance of the targeted invasive species through releases from intraspecific competition or cannibalism (Lewin et al. 2006). Consequently, it can be hypothesized that the removal of a nonrandom subset of individuals from the invading population by recreational angling might modify their population characteristics.

The intensive exploitation of wild populations selectively removes the most profitable individuals, that is, those that maximize the yield (Coltman et al. 2003; Belgrano and Fowler 2013), imposing “*unnatural”* selection for the less profitable individuals (Allendorf and Hard 2009). For instance, fishing typically involves the targeting of the larger individuals (Law 2000; Jørgensen et al. 2007), with the potential to affect subsequent reproductive success and recruitment (Biro and Post 2008). Changes in life-history traits can subsequently impact population structure and dynamics and lead to a drastic decline in the exploited stock or even local extinction (Law 2000; Allendorf et al. 2008; Palkovacs et al. 2012). Freshwater recreational angling is a significant component of the worldwide fishery (Cooke and Cowx 2004), but extant knowledge on the effects of angling-induced selection on wild and/or invasive populations remains relatively scarce (e.g., Cooke and Cowx 2006; Lewin et al. 2006; Philipp et al. 2009; Wilson et al. 2011). Recreational angling is usually size selective and orientated toward the capture of the largest individuals (Isermann et al. 2005; Cooke and Cowx 2006; Alós et al. 2008), and the higher catchability of larger individuals could be driven by their behavior (Biro and Post 2008; Uusi-Heikkilä et al. 2008) and/or by hook-size selectivity (Alós et al. 2008). A recent field-based study demonstrated recreational angling also selects individuals based on their morphological traits, leading to populations with smaller mouths and deeper bodies (Alós et al. 2014). In entirety, these findings indicate recreational angling can also act as an important human-induced source of selection (Philipp et al. 2009; Sutter et al. 2012).

**Figure 1:**
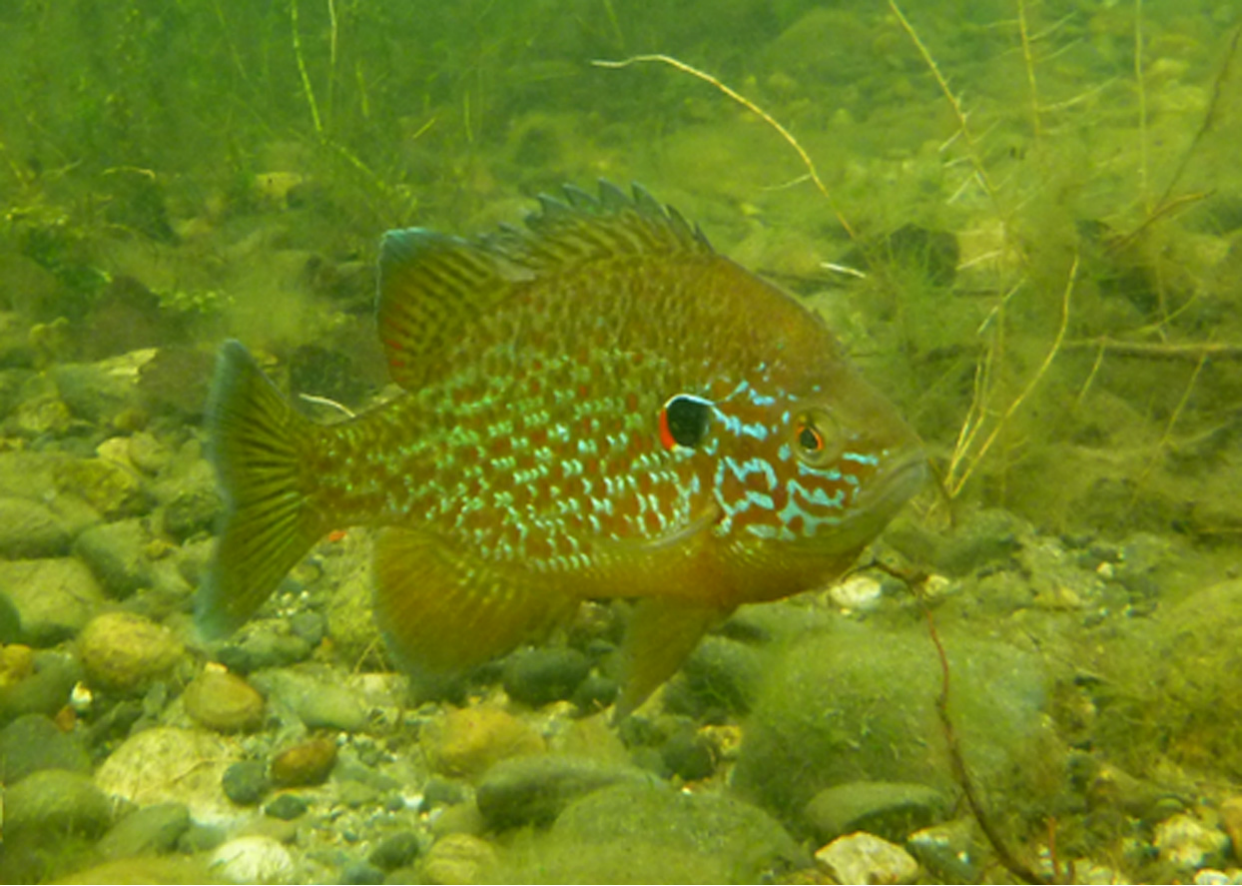
Male pumpkinseed (*Lepomis gibbosus*) nest guarding in a lake located in southwestern France (Photo: N. Charpin, EDB).

In this study, we tested whether fish removal by recreational angling impacts the population characteristics and juvenile growth rate (length at age and length increment) of an invasive freshwater fish species (*Lepomis gibbosus*; Fig.1) in 10 artificial lakes located in southwest France. In this country, *L. gibbosus* is one of the only two freshwater fish species that has a legal status of being “invasive” because of its potential for causing ecological disruption (Guevel 1997). This is important, as it means recreational anglers are legally required to remove any captured specimens and this is currently the only strategy in place for managing *L. gibbosus* in France. We therefore predicted that (1) populations with higher levels of removal pressure would comprise of smaller and lighter individuals through the largest individuals being more vulnerable to capture and so removal (Law 2000; Cooke and Cowx 2006; Jørgensen et al. 2007); (2) *L. gibbosus* populations in lakes with higher levels of removals would be of lower population abundance and biomass due to angling-induced mortality; (3) population abundance and biomass could increase under a certain minimal threshold of capture size, given that removal by angling is size selective; and (4) changes in growth rates of individuals will result from the increased risk of mortality that results from higher removal pressure and this would lead to either faster or slower growth rates (Enberg et al. 2012).

## Material and Methods

### Model species

Pumpkinseed *L. gibbosus* (L.) is a North American centrarchid that was introduced into Europe in the late 19th century and is now one of the most widely established introduced fishes in Europe (Garcia-Berthou and Moreno-Amich 2000a; Copp and Fox 2007). In its introduced range, *L. gibbosus* has been reported in both natural and artificial lentic ecosystems, and also some lotic ecosystems. In southern Europe, *L. gibbosus* populations display a high potential for invasiveness that is associated with high growth rate and fecundity (Copp and Fox 2007). Age and size at maturity varies between 1.0 and 3.9 years, and 61.4 and 96.2 mm, respectively (Copp and Fox 2007). Introduced *L. gibbosus* can also spawn several times within the same year (Fox and Crivelli 1997). The most common impact of *L. gibbosus* in its introduced range is diet overlap with native species and the consumption of eggs and molluscs (Garcia-Berthou and Moreno-Amich 2000a). Ontogenic diet shift is common in *L. gibbosus*, with individuals initially feeding on soft-bodied prey and then shifting toward hard-bodied prey (Garcia-Berthou and Moreno-Amich 2000b).

### Study area

The study was completed during 2012 in 10 artificial lakes localized along the flood plain of the Garonne River in southern Toulouse (southwestern France). These artificial lakes are former gravel pits (hereafter referred as lakes) that were located within a small geographic range (maximum distance between the lakes: 32.4 km) and with substrates dominated by a mix of sand, gravel, and pebble that are recognized as adequate habitats for *L. gibbosus*, notably for spawning (Kleef et al. 2008). In the studied lakes, fish species richness ranged from 5 to 15 (mean = 9.3 ± 1.1 SE), and the proportion of non-native species in the community ranged from 37.5 to 80.0% (mean = 54.6% ± 4.5 SE). Fish communities in the studied lakes were dominated by Cyprinidae (roach *Rutilus rutilus* and rudd *Scardinius erythrophthalmus*)*,* centrarchidae (*L. gibbosus* and largemouth bass *Micropterus salmoides*), and Percidae (perch *Perca fluviatilis* and pike-perch *Sander lucioperca*). Four obligate piscivorous fish species were sampled in these lakes: northern pike *Esox lucius*, *M. salmoides*, *P. fluviatilis,* and *S. lucioperca*.

### Fish sampling

*L. gibbosus* were sampled in the littoral habitat of each lake from mid-September to mid-October 2012 between 12:00 and 16:00 using point abundance sampling performed randomly by electrofishing (PASE, Nelva et al. 1979; Cucherousset et al. 2006b). This was completed from a boat along the shore to sample the shallow littoral habitat, that is, where the recreational angling primarily occurs (Cooke and Cowx 2004). The total number of points sampled per lake ranged from 20 to 45 (mean = 33.4 ± 2.1), depending upon lake size (smaller lakes used less sampling points) and covered the entire lake perimeter. All captured individuals were identified to species, and fork length (i.e., the length measured from the tip of snout to the fork of the tail) was estimated for all specimens (*FL*_e_, nearest 5 mm). Captured individuals of *L. gibbosus* were immediately euthanized using an overdose of eugenol, stored on ice and frozen in the laboratory (−20°C) until subsequent processing. After defrosting, subsamples of *L. gibbosus* (mean = 33 ± 1.69 individuals per population depending upon the total number of individuals sampled) were selected to encompass the full range of fork length and to be representative of the population size structure, with fish in each 10-mm-size class selected according to their proportion in the population. Fish were individually measured for fork length (*FL*_m_ ± 1 mm) and weighed (*W*_m_ ± 0.1 mg). Scales were removed below the lateral line and behind the dorsal fin, analyzed on a micro-projector (magnification: 48×) and aged through the counting of annual marks, with 25% subsets of scales (including scales of the oldest specimens) aged by a second operator for validation.

### Removal pressure and environmental characteristics

Removal pressure was quantified in each lake by counting the number of “coarse” anglers along the shoreline on 16 occasions during two consecutive years from April 2012 to March 2013 and from April 2013 to March 2014 so as to fully encompass the variability in angler numbers driven by weather conditions, seasons, and time. In the studied lakes, coarse anglers preferentially target cyprinid species over certain size thresholds using gears and hook sizes that also enable the capture of *L. gibbosus*. While the number of pumpkinseed actually captured by anglers was not directly estimated, it was assumed that the number of anglers was a direct proxy of removal pressure for *L. gibbosus*. Therefore, an index of removal pressure was calculated for each lake as the mean number of coarse anglers per shoreline length (expressed in number of anglers km^−1^). A set of environmental characteristics of lakes was then selected that were known as having strong influences on the population and individual characteristics of fish. Lake surface areas were estimated using GIS (km^2^), and lake productivity was quantified using the integrative trophic status index (TSI; <40 oligotrophic, 40–50 mesotrophic, 50–70 eutrophic, >70 hypereutrophic) calculated using chlorophyll a (*μ*g L^−1^), total phosphorus (*μ*g L^−1^), and Secchi (m) parameters (Carlson 1977). For each of these parameters, three replicate measurements were taken in each lake on 11th and 12th September 2012. Predation pressure was estimated as the catch per unit effort of obligate piscivorous fish species captured during electrofishing (ind PASE^−1^) in the littoral habitat of each lake. As these piscivorous fishes are gape-size limited predators and *L. gibbosus* are deep-bodied prey, a threshold of 95 mm was used as their minimum *FL*_e_ at which they could predate on young-of-the-year *L. gibbosus,* and thus, only predators with *FL*_e_ > 95 mm were used to quantify predation pressure.

### Data analyses

For each *L. gibbosus*, individual weight (*W*_e_ ± 0.1 mg) was estimated from the relationship between *FL*_m_ and *W*_m_ obtained in each lake and *FL*_e_. At the population level, *FL*_e_ and *W*_e_ were used to calculate the 90% quantile population fork length and 90% quantile population mass, respectively, to provide a representative estimate of the upper distribution of maximal length and mass at the population level. The total abundance of *L. gibbosus* was calculated as the catch per unit effort (CPUE defined as the number of individuals per PASE, ind PASE^−1^). In addition, the abundance of three different size classes of *L. gibbosus* (i.e., small bodied: *FL*_e_ < 60 mm, medium bodied: 60 mm ≤ *FL*_e_ < 90 mm and large bodied: *FL*_e_ ≥ 90 mm) was calculated (ind PASE^−1^). Finally, the biomass of *L. gibbosus* (g PASE^−1^) was estimated using *W*_e_. At the individual level, the back-calculated length at age was calculated using scale measurements and the scale proportional method (Francis 1990). Due to the lack of older individuals in several populations, length was back-calculated at age 1 and age 2. Protracted spawning (i.e., a second spawning event occurred late in summer) was revealed in a minority of lakes. This resulted in some very small young-of-the-year at the end of their first growth year (<20 mm). To remove the effect of such protracted spawning, juvenile growth rate was quantified here as the growth increment between the back-calculated lengths produced at ages 1 and 2 (Beardsley and Britton 2012).

### Statistical analyses

We first tested the temporal stability of removal pressure using a Spearman correlation between removal pressure obtained from April 2012 to March 2013 and from April 2013 to March 2014. Spearman correlations were then used to analyze the relationships between environmental characteristics (i.e., lake surface area, productivity, and predation pressure) and removal pressure. Population responses (i.e., fork length (90% quantile), mass (90% quantile), total abundance, abundance of small, medium, and large individuals, and biomass, *n *=* *10) to removal pressure were tested using linear models. As we predicted that the vulnerability to removal by angling was size selective, removal pressure might act nonmonotonically on population biomass and abundance below a certain capture size threshold. Therefore, linear models were performed to test the effect of removal pressure through angling on population biomass and abundance included both linear and quadratic terms to assess monotonic (linear and nonlinear) and nonmonotonic (hump-shaped and U-shaped) relationships. The quadratic term was removed when it was not significant according to a marginal t-test (Crawley 2007). Departures from homoscedasticity and the normality of residuals were assessed graphically using Tukey Ascombe plots and Q-Q plots, respectively. Fisher and Shapiro–Wilk tests were used to confirm the assumption of homoscedasticity, and normality and appropriate transformations were performed when necessary. Specifically, the 90% quantile fork length, the 90% quantile mass, population biomass, total abundance, and abundance of small-bodied individuals were log-transformed. Removal pressure was square-root transformed to ensure a more even dispersion of lakes.

To test the effects of removal pressure and environmental characteristics on individual length at age 1 and 2 and juvenile growth rate, linear mixed effects models were performed with lake as a random factor (Pinheiro et al. 2012). All models included removal pressure, lake productivity, predation pressure, and *L. gibbosus* total abundance as fixed effects. These last three variables were selected to encompass the main drivers of individual variability within a population (namely resource availability, predation and competition, respectively; Araújo et al. 2011). Spearman correlation matrices were performed to detect potential collinearity between predictors, and when collinearity was presented, the residuals from the relationship between the two predictors were used in the linear mixed effects models (Zuur et al. 2009), resulting in the residuals from the relationship between removal pressure and lake productivity being used in the final linear mixed effects models. Lengths at age 1 and age 2 were log-transformed to fit a normal distribution, and individual age (categorical variable) was included in the models to account for a potential cohort effect. Length at age 1 was included in the model with the juvenile growth rate to account for the fact that fish growth was not isometric. All analyses that were run with a full model included one-way interactions between removal pressure and predation pressure, *L. gibbosus* total abundance and lake productivity. Interactions were subsequently removed when nonsignificant using backward selection based on marginal t-test (Crawley 2007). For each final linear mixed effects model, the assumption of homoscedasticity and normality of residuals were checked using graphical tools and the residuals were plotted against each explanatory variable to check for independence. All linear mixed effects models conformed to the assumptions. All statistical analyses were performed using R (R Development Core Team 2013). Where error around the mean is expressed, it represents standard error.

## Results

### Removal pressure through angling and environmental characteristics of lakes

Removal pressure through angling was highly variable across the 10 lakes, ranging from 0.06 to 1.94 anglers per km of shoreline (mean = 0.71 ± 0.20 anglers km^−1^, Table1). Removal pressure measured from April 2012 to March 2013 and from April 2013 to March 2014 were significantly and positively correlated (*r* = 0.76, *S* = 40, 8 df, *P *=* *0.016), indicating a strong temporal stability of removal pressure in each lake across years. Each year, the level of removal pressure peaked from April to September, overlapping with the spawning period of *L. gibbosus* when individuals are more vulnerable to removal through angling because of nest guarding behaviors. Lake surface area ranged from 0.018 to 0.208 km^2^ (mean = 0.118 ± 0.023 km^2^, Table1) and was not significantly correlated to removal pressure (*r* = −0.28, *S* = 212, 8 df, *P *=* *0.427). Lake productivity ranged from nearly mesotrophic to hypereutrophic, with TSI ranging from 52.97 to 72.16 (mean = 60.52 ± 2.15, Table1) and was significantly and positively correlated to removal pressure (*r* = 0.67, *S* = 54, 8 df, *P *=* *0.039). Predation pressure by piscivorous fish species ranged from 0.13 to 2.71 ind PASE^−1^ (mean = 0.79 ± 0.24 ind PASE^−1^, Table1) and was not significantly correlated to removal pressure (*r* = 0.12, *S* = 145.94, 8 df, *P *=* *0.751).

**Table 1:** Environmental characteristics of the ten studied lakes monitored from April 2012 to March 2014

Site code	1	2	3	4	5	6	7	8	9	10
Removal pressure (anglers km^−1^)	0.06	0.16	0.28	0.33	0.35	0.42	1.09	1.25	1.26	1.94
Surface area (km^2^)	0.03	0.18	0.09	0.21	0.19	0.21	0.10	0.04	0.12	0.02
Productivity (TSI)	55.47	59.22	52.97	54.03	57.83	65.19	54.20	66.88	72.16	67.29
Fish species diversity	5	5	6	12	15	11	8	8	13	10
Predation (ind PASE^−1^)	0.49	0.13	0.32	0.89	0.89	2.71	1.34	0.40	0.18	0.50
Abundance (ind PASE^−1^)	0.84	2.81	0.53	4.24	1.50	0.74	1.79	7.52	1.25	5.93
Biomass (g PASE^−1^)	30.46	14.27	9.01	8.21	20.57	5.52	4.60	24.03	11.48	42.11

### Population responses to removal pressure through angling

**Figure 2:**
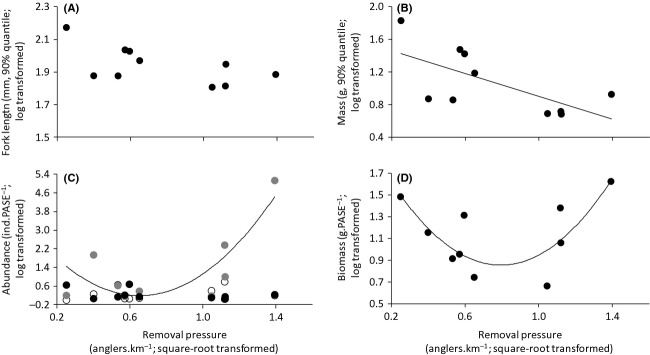
Relationship between removal pressure through angling (anglers km^−1^, square-root transformed) and (A) fork length (mm, 90% quantile, log-transformed); (B) mass (g, 90% quantile, log-transformed); (C) abundance of small-bodied (*FL*_e_ < 60 mm, open dots), medium-bodied (60 mm ≥ *FL*_e_ < 90 mm, gray dots), and large-bodied (*FL*_e_ ≥ 90 mm, solid dots) individuals (ind PASE^−1^); and (D) biomass (g.PASE^−1^, log-transformed) across studied populations (*n* = 10). Significant relationships are depicted with solid lines. For panel (C), the abundance of small-bodied individuals is log-transformed and the significant relationship is between removal pressure and the abundance of medium-bodied individuals.

The 90% quantile population fork length was not significantly related to removal pressure (*R*^2^ = 0.37, *F*_1,8_ = 4.69, *P *=* *0.062; Fig.2A, Table2) while the 90% quantile population mass was significantly and negatively related to removal pressure (*R*^2^ = 0.44, *F*_1,8_ = 6.30, *P *=* *0.036; Fig.2B, Table2), revealing that maximal individual mass in the population decreased as removal pressure increased. The total abundance of *L. gibbosus* varied over a 14-fold range across lakes, but was not significantly related to removal pressure (*R*^2^ = 0.25, *F*_1,8_ = 2.63, *P *=* *0.144; Table2). *L. gibbosus* populations were mainly composed of medium-bodied individuals (mean proportion = 44.2 ± 8.6%, Fig.2C), whereas the mean proportion of small- and large-bodied individual represented 35.0% (±10.1) and 20.8% (±8.0) of the total abundance, respectively. The abundance of small-bodied and large-bodied individuals did not vary significantly with removal pressure (*R*^2^ = 0.07, *F*_1,8_ = 0.60, *P *=* *0.460 and *R*^2^ = 0.18, *F*_1,8_ = 1.76, *P *=* *0.222, respectively; Fig.2C, Table2). However, the abundance of medium-bodied individuals was significantly related to removal pressure in a nonmonotonic manner (*R*^2^ = 0.70, linear term: *F*_1,7_ = 9.34, *P *=* *0.019, quadratic term: *F*_1,7_ = 7.11, *P *=* *0.032; Table2). Specifically, the abundance of medium-bodied individuals was higher at low and high levels of removal pressure (Fig.2C). The biomass of *L. gibbosus* also varied considerably among populations, ranging between 3.28 and 42.11 g PASE^−1^ (mean = 17.0 ± 3.9 g PASE^−1^). A significant U-shaped relationship between removal pressure and population biomass was detected (*R*^2^ = 0.60, linear term: *F*_1,7_ = 0.33, *P *=* *0.583, quadratic term: *F*_1,7_ = 10.05, *P *=* *0.016, Table2), with population biomass being lower at intermediate levels of removal pressure (Fig.2D).

**Table 2:** Results of the simplified regression models assessing linear and quadratic relationships between removal pressure through angling and population responses (90% quantile fork length (mm); 90% quantile mass (g); total abundance (ind PASE^−1^); abundance of small-bodied (*FL*_e_ < 60 mm), medium-bodied (60 mm ≥ *FL*_e_ < 90 mm), and large-bodied individuals (FL ≥ 90 mm) (ind PASE^−1^); and biomass (g PASE^−1^); *n* = 10). Significant *P*-values are displayed in bold

Response variables	Source of variation	df	Estimate (SE)	*F*	*P*
Fork length (90% quantile)	Removal pressure	8	−0.18 (0.09)	4.69	0.062
	Intercept	8	2.08 (0.07)		
Mass (90% quantile)	Removal pressure	8	−0.70 (0.28)	6.30	**0.036**
	Intercept	8	1.60 (0.24)		
Total abundance	Removal pressure	8	0.52 (0.32)	2.63	0.143
	Intercept	8	−0.12 (0.27)		
Small-bodied abundance	Removal pressure	8	0.19 (0.25)	0.60	0.460
	Intercept	8	0.11 (0.21)		
Medium-bodied abundance	Removal pressure	7	−9.92 (4.78)	9.14	**0.019**
	Removal pressure^2^	7	7.63 (2.86)	7.11	**0.032**
	Intercept	7	3.42 (1.72)		
Large-bodied abundance	Removal pressure	8	−0.26 (0.20)	1.76	0.222
	Intercept	8	0.44 (0.17)		
Biomass	Removal pressure	7	−3.42 (1.13)	0.33	0.583
	Removal pressure^2^	7	2.15 (0.68)	10.05	**0.016**
	Intercept	7	2.21 (0.41)		

### Individual responses to removal pressure through angling

At the individual level, none of the tested environmental parameters (i.e., productivity, predation pressure and abundance) had a significant effect on length at age 1 (*F*_1,196_ = 1.19, *P *=* *0.278; *F*_1,6_ = 2.14, *P *=* *0.194; *F*_1,6_ = 1.18, *P *=* *0.320; Table3, Table A1 in the Appendix S1), length at age 2 (*F*_1,145_ = 0.18, *P *=* *0.675; *F*_1,6_ = 0.09, *P *=* *0.774; *F*_1,6_ = 0.75, *P *=* *0.419; Table3, Table A1 in the Appendix S1), and juvenile growth rate (*F*_1,147_ = 1.70, *P *=* *0.194; *F*_1,6_ = 0.03, *P *=* *0.877; *F*_1,6_ = 0.56, *P *=* *0.482; Table3, Table A2 in the Appendix S1). No significant relationship between removal pressure and the length at age 1 was found (*F*_1,6_ = 0.58, *P *=* *0.476; Table3, Table A1 in the Appendix S1) whereas length at age 2 was significantly and negatively affected by removal pressure (*F*_1,6_ = 8.62, *P *=* *0.026; Table3, Table A1 in the Appendix S1). We also found that length at age 1 had a significant and negative effect on juvenile growth rate (*F*_1,147_ = 33.68, *P *<* *0.001; Table3, Table A2 in the Appendix S1), indicating that individuals with a larger body size at age 1 subsequently grew proportionally slower than individuals with smaller body size at age 1. Finally, juvenile growth rate was significantly and negatively affected by removal pressure (*F*_1,6_ = 6.37, *P *=* *0.045; Table3, Table A2 in the Appendix S1).

**Table 3:** Summary of the linear mixed effects models used to test for the effects of removal pressure through angling and environmental characteristics (productivity, predation pressure, and abundance) on length at age 1 (*n* = 211), length at age 2 (*n* = 159), and juvenile growth rate (*n* = 159). Productivity refers to the residuals from the relationship between removal pressure and lake productivity. Estimate ± SE are reported, and significant *P*-values are displayed in bold. Further details available in the Supporting Information

	Length at age^1^	Length at age^2^	Juvenile growth rate
Age^1^ *or* FL at age 1^2^	*	*	−23.11 ± 3.95
	*P *=* ***0.013**	*P *=* *0.107	*P ***<*** ***0.001**
Removal pressure	0.09 ± 0.07	−0.08 ± 0.04	−10.34 ± 6.05
	*P *=* *0.476	*P *=* ***0.026**	*P *=* ***0.045**
Productivity (res.)	−0.01 ± 0.01	<0.01 ± <0.01	0.49 ± 0.42
	*P *=* *0.278	*P *=* *0.675	*P *=* *0.194
Predation	0.04 ± 0.03	<0.01 ± 0.02	−0.12 ± 2.62
	*P *=* *0.194	*P *=* *0.774	*P *=* *0.877
Abundance	−0.09 ± 0.08	−0.04 ± 0.02	−4.67 ± 6.23
	*P *=* *0.320	*P *=* *0.419	*P *=* *0.482
Intercept	1.23 ± 0.07	1.92 ± 0.03	87.37 ± 7.22
	*P ***<*** ***0.001**	*P ***<*** ***0.001**	*P ***<*** ***0.001**

1 Age was included in the models with length at age.

2 FL at age 1 was included in the models with growth rate.

* Not available as age was a categorical variable.

## Discussion

Human activities have been widely recognized as a driver of rapid trait change in wild animal populations (Hendry et al. 2008; Darimont et al. 2009; Palkovacs et al. 2012), affecting individuals in a nonrandom manner (Coltman et al. 2003). However, the impacts of recreational angling on fish populations are usually underappreciated (Arlinghaus et al. 2010) and this is particularly true for the cases where angling is used as a removal method to extirpate or regulate invasive fish species. In the present study, we observed several modifications of population and individual characteristics of an invasive fish species driven by removal pressure through angling. We demonstrated that removal pressure might induce body mass truncation, with populations under high removal pressure mainly composed of lighter individuals, while the effects on individual maximal length were not significant. Contrary to our predictions, removal pressure in the present study did not affect the total abundance of invasive *L. gibbosus*. However, the abundance of medium-bodied individuals and population biomass were modified in a nonmonotonic manner. Finally, we found that removal pressure decreased length at age 2 of individuals. Changes of individual length at age could be linked to difference in juvenile growth trajectories, and we found a significant and negative effect of removal pressure on juvenile growth rate.

Exploitation of fish populations can induce selective mortality (Law 2000; Lewin et al. 2006; Belgrano and Fowler 2013). Here, selective harvesting was most likely occurring through the removal of larger individuals, as maximal individual mass in the populations decreased with increasing removal pressure. Such truncation in mass could potentially explain the observed increase in the abundance of medium-bodied individuals at high removal pressure through competitive release from the larger individuals (Lewin et al. 2006). Recreational angling gears are size selective, with hook size being an important driver of captured individual size (Alós et al. 2008). As anglers in the study area are not specifically targeting *L. gibbosus*, it is unlikely that they modify hook size (and therefore minimal capture size) as *L. gibbosus* length at age decreased. Consequently, the increase abundance of medium-bodied individuals could also be explained by the decrease in length at age because populations at high removal pressure were mostly composed of individuals included in smaller size classes that are less vulnerable to capture by angling and more adept at avoiding anglers' hooks. The U-shaped relationship observed between removal pressure and population biomass was probably driven by the removal of larger individuals with increasing removal pressure that was compensated by the increase abundance of medium-bodied individuals at high removal pressure. Consequently, populations subjected to high selective removal through angling become dominated by smaller individuals and are potentially more sensitive to changes in environmental conditions and more subject to unstable dynamics (Anderson et al. 2008), revealing the need to temporally integrate the effects of selective removal pressure on invasive fish populations.

Decreases in length at age and in juvenile growth rate could reflect a shift toward a slower life-history phenotype as removal pressure increased and therefore be a potential mechanism to reduce their vulnerability of capture (Conover and Munch 2002; Biro and Post 2008). Nevertheless, juvenile growth rate in harvested fish populations often increases due to the release in intraspecific competition and the improvement of resource access (Fenberg and Roy 2008; Enberg et al. 2012). Here, the abundance of small-bodied individuals was not affected by removal pressure and we also observed a high abundance of medium-bodied individuals in sites with high level of removal pressure. This suggested that intraspecific competition probably persisted in the studied lakes and might have potentially dampened the selection for faster juvenile growth rate. Such changes in life-history trajectories related to angling pressure can be driven by phenotypic plasticity or natural selection (Palkovacs et al. 2012; Van Wijk et al. 2013), and this remains to be tested to fully appreciate the evolutionary consequences of selective removal on invasive populations.

The existence of compensatory mechanisms is a common phenomenon in harvested wild populations and can notably lead to changes in reproductive investment (e.g., Lewin et al. 2006). Angling has been demonstrated to lead to an earlier age and size at maturity, with mature individuals potentially increasing their reproductive investment (Jørgensen et al. 2007; Fenberg and Roy 2008). In the present study, reduced length at age 2 is likely to decrease size at maturity. Indeed, the observed length at age 2 (mean = 70.1 mm ± 1.2 SE) was included within the range of size at maturity reported in the literature for invasive populations of *L. gibbosus* in Europe (Copp and Fox 2007; Cucherousset et al. 2009). Reduced size at maturity could lead to a decline in fecundity and fitness (Walsh et al. 2006), with potential negative effects on fish population abundance. However, under stress conditions, multiple and protracted spawning strategies could be adopted in *L. gibbosus* (Garvey et al. 2002) to counterbalance the negative effects of earlier size at maturity. Here, protracted spawning was observed in the two lakes that had the highest removal pressure, suggesting that recreational angling potentially modifies the reproductive strategy of *L. gibbosus*. Thus, further studies should aim at quantifying the impacts of selective removal on reproductive strategies and its ultimate consequences on population dynamics.

In the context of biological invasions, management methods used to control invasive populations are widely employed in lakes, where total eradication is either relatively inefficient or almost impossible to achieve (Britton et al. 2011). While the current removal strategy aims at reducing the abundance of invasive populations through angling, its real efficiency as a control method is questionable as the total abundance of populations did not decrease significantly as removal pressure increased. Instead, we found that the abundance of medium-bodied individuals was higher at high removal pressure. Removal selection against larger individuals leads to populations composed of smaller individuals with reduced mouth gape, which could strongly alter the prey–predators relationships and by extension reshape ecological interactions between organisms and food webs (Shackell et al. 2009; Palkovacs et al. 2012; Audzijonyte et al. 2013; Fraser 2013). Here, higher abundances of medium-bodied individuals could lead, for instance, to increased predation pressure on some specific invertebrate taxa (i.e., chironomids and heteroptera) and higher interspecific competition, with potential for cascading consequences. Overall, the current selective methods to control invasive species do not appear particularly successful, suggesting the development of more proactive strategies and the use of less selective methods.

Human-induced environmental disturbances are a major agent of rapid evolution in wild populations (Hendry et al. 2008; Darimont et al. 2009) that affect individuals in a nonrandom manner (Coltman et al. 2003). Variability of individuals within a population drives difference in vulnerability to angling (Uusi-Heikkilä et al. 2008). Therefore, in a context of biological invasions, further studies need to investigate the correlated traits (i.e., genetic, morphologic, trophic, physiologic, and behavior) involved in the nonrandom removal of individuals to fully appreciate the potential ecological and evolutionary consequences of the management of invasive species.

## Supporting Information

**Appendix S1.** Results of the linear mixed effects models used to test for the effects of removal pressure and environmental characteristics on invasive *Lepomis gibbosus*.
