# Supplementary material for: Impacts of invasive fish removal through angling on population characteristics and juvenile growth rate
Source: Ecol Evol. 2015 May 9;5(11):2193–202. doi: 10.1002/ece3.1471 (PMC4461421; doi:10.1002/ece3.1471)
Supplement: Supplementary file 1 [file ece30005-2193-sd1.docx]

**Supporting Information**

**Impacts of invasive fish removal through angling on population characteristics and juvenile growth rate**

C. Evangelista, J.R. Britton, J. Cucherousset^1*^

^*^ Corresponding author: *julien.cucherousset@univ-tlse3.fr*

**Appendix S1: Results of the linear mixed effects models used to test for the effects of removal pressure and environmental characteristics on invasive *Lepomis gibbosus*.**

**Table A1:** Results of the linear mixed effects models used to test for the effects of removal pressure through angling and environmental characteristics (productivity, predation pressure and abundance) on length at age 1 (n = 211; marginal R-squared = 0.05 and conditional R-squared = 0.54) and length at age 2 (n = 159; marginal R-squared = 0.10 and conditional R-squared = 0.58). Productivity refers to the residuals from the relationship between removal pressure and lake productivity. Significant P-values are displayed in bold.

|  | **Length at age 1** | | | |  | **Length at age 2** | | | |
| --- | --- | --- | --- | --- | --- | --- | --- | --- | --- |
|  | **Df** | **Estimate (SE)** | **F** | ***P*** |  | **Df** | **Estimate (SE)** | **F** | ***P*** |
| **Fixed effects** |  |  |  |  |  |  |  |  |  |
| Age | 4,196 | * | 3.25 | **0.013** |  | 3,145 | * | 2.07 | 0.107 |
| Angling pressure | 1,6 | 0.09 (0.07) | 0.58 | 0.476 |  | 1,6 | - 0.08 (0.04) | 8.62 | **0.026** |
| Productivity (residuals) | 1,196 | - 0.01 (0.01) | 1.19 | 0.278 |  | 1,145 | < 0.01 (< 0.01) | 0.18 | 0.675 |
| Predation | 1,6 | 0.04 (0.03) | 2.14 | 0.194 |  | 1,6 | < 0.01 (0.02) | 0.09 | 0.774 |
| Abundance | 1,6 | - 0.09 (0.08) | 1.18 | 0.320 |  | 1,6 | - 0.04 (0.02) | 0.75 | 0.419 |
| Intercept | 1,196 | 1.23 (0.07) | 3157.95 | **< 0.001** |  | 1,145 | 1.92 (0.03) | 22340.35 | **< 0.001** |
| **Random effect** |  | **Variance (SD)** |  |  |  |  | **Variance (SD)** |  |  |
| Intercept |  | < 0.01 (0.05) |  |  |  |  | < 0.01 (0.03) |  |  |
| Residuals |  | 0.05 (0.23) |  |  |  |  | 0.01 (0.08) |  |  |

^*^ not available since Age was a categorical variable

**Table A2**: Results of the linear mixed effects model used to test for the effects of removal pressure and environmental characteristics (productivity, predation pressure and abundance) on juvenile growth rate (n = 159; marginal R-squared = 0.17 and conditional R-squared = 0.63). Productivity refers to the residuals from the relationship between removal pressure and lake productivity. Significant P-values are displayed in bold.

|  |  | **Growth rate** | | | |
| --- | --- | --- | --- | --- | --- |
|  |  | **Df** | **Estimate (SE)** | **F** | ***P*** |
| **Fixed effects** |  |  |  |  |  |
| FL at age-1 |  | 1,147 | - 23.11 (3.95) | 33.68 | **< 0.001** |
| Angling pressure |  | 1,6 | - 10.34 (6.05) | 6.37 | **0.045** |
| Productivity (residuals) |  | 1,147 | 0.49 (0.42) | 1.70 | 0.194 |
| Predation |  | 1,6 | - 0.12 (2.62) | 0.03 | 0.877 |
| Abundance |  | 1,6 | - 4.67 (6.23) | 0.56 | 0.482 |
| Intercept |  | 1,147 | 87.37 (7.22) | 627.67 | **< 0.001** |
| **Random effect** |  |  | **Variance (SD)** |  |  |
| Intercept |  |  | 26.65 (5.16) |  |  |
| Residuals |  |  | 112.29 (10.60) |  |  |
